# Supplementary material for: A fault-intrusion-tolerant system and deadline-aware algorithm for scheduling scientific workflow in the cloud
Source: PeerJ Comput Sci. 2021 Nov 2;7:e747. doi: 10.7717/peerj-cs.747 (PMC8576554; doi:10.7717/peerj-cs.747)
Supplement: Supplemental Information 6 [file peerj-cs-07-747-s006.zip › Myproject1.1/docs/org/cloudbus/cloudsim/class-use/ParameterException.html]

Uses of Class org.cloudbus.cloudsim.ParameterException


JavaScript is disabled on your browser.


- Overview
- Package
- Class
- Use
- Tree
- Deprecated
- Index
- Help

- Prev
- Next

- Frames
- No Frames

- All Classes

## Uses of Class org.cloudbus.cloudsim.ParameterException

- Packages that use ParameterException

  | Package | Description |
  | --- | --- |
  |  |  |
  | --- | --- |
  | org.cloudbus.cloudsim |  |
  | org.workflowsim |  |
- - ### Uses of ParameterException in org.cloudbus.cloudsim

    Constructors in org.cloudbus.cloudsim that throw ParameterException

    Constructor and Description

    `File(File file)`

    Copy constructor, i.e.

    `File(java.lang.String fileName,
    int fileSize)`

    Creates a new DataCloud file with a given size (in MBytes).

    `FileAttribute(java.lang.String fileName,
    int fileSize)`

    Allocates a new FileAttribute class.

    `HarddriveStorage(double capacity)`

    Creates a new harddrive storage with a given capacity.

    `HarddriveStorage(java.lang.String name,
    double capacity)`

    Creates a new harddrive storage with a given name and capacity.

    `SanStorage(double capacity,
    double bandwidth,
    double networkLatency)`

    Creates a new SAN with a given capacity, latency, and bandwidth of the network connection.

    `SanStorage(java.lang.String name,
    double capacity,
    double bandwidth,
    double networkLatency)`

    Creates a new SAN with a given capacity, latency, and bandwidth of the network connection.
  - ### Uses of ParameterException in org.workflowsim

    Constructors in org.workflowsim that throw ParameterException

    Constructor and Description

    `ClusterStorage(java.lang.String name,
    double capacity)`

    Initialize a ClusterStorage

- Overview
- Package
- Class
- Use
- Tree
- Deprecated
- Index
- Help

- Prev
- Next

- Frames
- No Frames

- All Classes
